# Chronic Pain in a Modern Virally Suppressed HIV Cohort: Associations with Comorbidities Depression and Disability

**DOI:** 10.21203/rs.3.rs-6631567/v1

**Published:** 2025-06-24

**Authors:** Ronald J. Ellis, Robert K. Heaton, J. Hampton Atkinson, Murray Stein, Crystal Wang, Tyler R. Bell, Andrew Miller, David Grelotti, David Moore

**Affiliations:** University of California San Diego; University of California San Diego; University of California San Diego; University of California San Diego; University of California San Diego; University of California San Diego; Emory University; University of California San Diego; University of California San Diego

**Keywords:** depression, mood, chronic pain, neuropathic pain, opioid use

## Abstract

Chronic pain (CP) is common among people with HIV (PWH), yet its prevalence and associated factors in those receiving modern, virally suppressive antiretroviral therapy (ART) are not well understood. This prospective observational study compared CP frequency and associated outcomes between PWH and people without HIV (PWoH). Participants (40 PWH, 23 PWoH) completed a questionnaire assessing daily pain lasting more than three months. Additional data included pain intensity, interference with daily activities, opioid use, and depressed mood (Beck Depression Inventory-II), as well as HIV clinical markers and comorbidities. Groups were demographically similar; all PWH were virally suppressed, with a median HIV duration of 30.6 years, nadir CD4 count of 300 cells/μL, and current CD4 count of 644 cells/μL. CP was significantly more frequent in PWH (60%) than in PWoH (22%; OR = 5.4 [1.67, 17.5]; p = 0.0028). Among PWH, CP was associated with greater daily activity interference, higher opioid use (38% vs. 6%), and increased neuropathic pain symptoms. PWH with CP also had higher BDI-II scores, indicating worse mood. These findings suggest that CP remains prevalent and disabling among PWH despite effective modern-day ART, underscoring the need for targeted pain assessment and management in this population.

## Introduction

Chronic pain (CP) is a frequent and debilitating condition in the general population, with significant implications for quality of life. Despite increasing attention to CP in general, it remains understudied in people with HIV (PWH), as highlighted in a recent publication from the Global Task Force on Chronic Pain in People with HIV. CP affects an estimated 39–85% of PWH, compared to 20–30% of the general population,^1–4^ and is associated with substantial disability in PWH, affecting various aspects of well-being.^5,6^ While numerous reviews have been published recently,^7–9^ there is a dearth of primary data on CP’s frequency, causes, and associated factors in virally suppressed PWH on modern antiretroviral therapy (ART). Another issue unique to PWH is the frequent co-occurrence of multiple chronic pain disorders.^1,3,4^ CP can also influence treatment adherence,^7,10^ retention in care,^8,11^ and virologic outcomes in PWH.^12^

Given the potential implications for this population’s poor quality of life and treatment outcomes, these gaps in understanding chronic pain in PWH are highly clinically significant.^13^ The field needs to advance its understanding of the frequency of chronic pain in virally suppressed PWH and its associations with comorbidities such as peripheral neuropathy^14^ and other factors in PWH on modern virally suppressive ART regimens.

CP in PWH is often underdiagnosed and undertreated;^15^ few evidence-based management options are available. Many healthcare providers lack knowledge about HIV-related pain syndromes and effective pain management. Other barriers to pain management include lack of access to specialized pain services and the unavailability or high cost of pain medications^16,17^. Pain management is also complicated because PWH suffer from high rates of opioid use disorders,^18,19^ often involving self-medicating by inappropriately using medications or substance use.^20,21^

Depression, a common comorbidity in PWH, further complicates the scenario. Extensive links between chronic pain and depressed mood have been demonstrated in the general population but are underexplored in PWH.^21–23^ Shared biological and anatomical pathways that link chronic pain to depressed mood are amplified in PWH, potentially explaining the increased frequency of both conditions in this group. These shared pathways include chronic inflammation – which persists even in virally suppressed PWH; the brain reward system, comprising mesolimbic dopaminergic projections and other pathways compromised by HIV, central sensitization, and alterations in glutamatergic neurotransmission.^24^ Specific brain regions implicated in depressed mood and chronic pain include the dorsolateral prefrontal cortex and the anterior and posterior cingulate cortices.^25,26^ Socio-environmental factors to which PWH are vulnerable, such as social isolation, stigma, and financial hardship, are also associated with chronic pain and depressed mood.^27^

Our overarching objective regarding chronic pain was to respond to the emerging literature on the many chronic comorbidities that result in poor quality of life and daily functioning in older PWH, including chronic pain. We sought to characterize CP in a modern cohort of virally suppressed PWH, compare it to CP in demographically comparable PWoH, and assess its risk factors, particularly comorbidities, and its impact on clinical outcomes and quality of life. For the primary comparison, we hypothesized that chronic pain would be more common in PWH than in PWoH. Secondary, exploratory comparisons evaluated the patterns and sources of CP, risk factors for CP, and the associations of chronic pain with various characteristics, including opioid use, interference in daily activities, and impact on clinical outcomes and quality of life. We designed this as an exploratory analysis to generate hypotheses for future research. For the primary comparison, we hypothesized that chronic pain would be more common in PWH than in PWoH.

## Methods

### Participants

We studied PWH and PWoH in the HIV Neurobehavioral Research Center (HNRC) cohort evaluated between January 2022 and May 2023. Chronic pain is one of the many comorbidities studied within the HNRC, which maintains a cohort of well-characterized participants to facilitate research into the neurobehavioral consequences of HIV. In 2022, in response to increasing community concern and growing research evidence about the significant impact of chronic pain on people with HIV, we added a chronic pain assessment for all participants. This work supports the HNRC’s overarching goal of providing data, biospecimens, and expertise to address the many health challenges faced by PWH. Inclusion criteria were age 21 years or over and with or without HIV infection documented by serologic testing. Participants with active conditions that might interfere with study participation or interpretation of data collected from them (e.g., current intoxication) were excluded. Relevant sociodemographic variables, such as age, sex, education, and socioeconomic status, were collected for all participants. The study was approved by the local Ethics Committee of the University of California San Diego. and was conducted in accordance with the Declaration of Helsinki. All participants gave informed consent for their participation in the study.

## Measures

### Chronic pain questionnaire.

To evaluate chronic pain and its relation to depressed mood and functional outcomes, we administered a questionnaire that operationalizes the IASP/ICD-11 diagnostic criteria.^28^ While not formally validated, it directly maps to IASP classifications for: (1) pain duration and frequency, (2) pain intensity and interference, (3) pain source categories, and (4) severity specifiers. The complete questionnaire is provided in Supplementary Appendix A to facilitate replication and future validation studies. Chronic pain was defined as daily or almost daily pain lasting over 3 months. Those who reported chronic pain answered additional questions about the intensity of their pain (What is the average intensity of your pain? Using the line below, tell us the number of your pain intensity between 0 and 100), interference in daily activities (In the past 90 days, how much has your pain interfered with doing your normal daily activities - e.g., physical activity including exercise, work, caring for your family, housework), use of pain medications, and pain source. Following the IASP/ICD-11 taxonomy, we use ‘pain source’ rather than ‘pain location’ to capture both the subjective experience and clinical assessment of pain origin. This classification integrates anatomical regions (e.g., back) and body systems (e.g., neuropathic) to reflect clinical complexity, particularly important for conditions where experienced pain location may differ from its source. Because pain in HIV affects multiple body systems,^14^ we examined a range of pain sources, including musculoskeletal, postsurgical, post-traumatic, neuropathic, and back and leg pain. To identify these, participants were asked, “What types of daily or almost daily pain did your doctor say that you had, or do you believe you have (please check all that apply)?”

### Depressed mood and other psychiatric characteristics.

Current mood symptoms were evaluated with the Beck Depression Inventory-II (BDI-II), which is a 21-item self-report instrument. Each item is rated on a 4-point Likert scale from 0 to 3 (worst).^29^ BDI-II total score ranges from 0 to 63. Component BDI-II subscales capture cognitive, somatic, affective, and apathy symptoms.^30–32^ Anxiety was characterized by the Overall Anxiety Severity and Impairment Scale (OASIS), a brief 5-item self-report instrument designed to assess the severity and functional impairment associated with symptoms of anxiety across different disorders. The OASIS has demonstrated strong psychometric properties such as unidimensional factor structure, excellent internal consistency, and good convergent and discriminant validity.^33^ Lifetime psychiatric and substance disorders were characterized using the Composite International Diagnostic Interview (CIDI),^34^ a cross-cultural assessment of alcohol, drug use, and mental disorders designed for use by trained lay interviewers.

### Medical characteristics.

HIV disease was diagnosed by enzyme-linked immunosorbent assay with Western blot confirmation. Routine clinical chemistry panels, complete blood counts, rapid plasma reagin, hepatitis C virus antibody positivity, and CD4 + T cells (flow cytometry) were performed. Levels of HIV viral load in plasma were measured using reverse transcriptase-polymerase chain reaction (Amplicor, Roche Diagnostics, Indianapolis, IN), with a lower limit of quantitation (LLQ) of 50 copies/ml. HIV viral load was dichotomized as detectable vs. undetectable at the LLQ of 50 copies/ml. CD4 nadir was assessed by self-report. All participants completed a substance use history that included current opioid use. Opioid use was evaluated at the time of study evaluation through self-report and urine toxicology testing. Both prescribed medications and illicit use were captured, with use classified as present or absent based on either positive self-report or positive toxicology result.

Detailed medical and neurological histories and concomitant medications including antidepressants and antiretroviral (ARV) drug exposure history were captured via a structured, clinician-administered questionnaire. Medical comorbidities were comprehensively evaluated through clinician-administered medical history and laboratory studies, including liver function testing, complete blood counts, and a comprehensive metabolic panel.

### Clinical assessment of neuropathy and neuropathic pain.

Because sensory polyneuropathy and neuropathic pain are common sources of chronic pain in PWH, we performed targeted interviews and neurological examinations to characterize these. Centrally trained clinicians (mid-level practitioners and physicians) conducted standardized, validated evaluations.^35,36^ They included clinical examination for neuropathy signs (bilateral distal vibration, sharpness, and touch loss in the legs and feet, and reduced ankle reflexes) and self-reported neuropathic pain. Distal sensory polyneuropathy (DSP) was defined as the presence of two or more of these signs bilaterally. Distal neuropathic pain (DNP) was defined as burning, aching, or shooting symptoms in the distal legs and feet and was classified into five grades of clinician-rated pain severity distal sensory polyneuropathy based on participant reports: none, slight (occasional, fleeting), mild (frequent), moderate (frequent, disabling) and severe (constant, daily, disabling, requiring analgesic medication or other pain medication).^29^

*Quality of life* was assessed using the Medical Outcomes Study HIV Health Survey Short Form 36 (MOS-HIV SF-36), a reliable and valid tool for assessing overall quality of life, daily functioning, and physical health.^37^ The MOS-HIV contains 36 questions that assess various physical and mental dimensions of health. Items are grouped into two categories (Physical and Mental Health), with 9 subcategories (Physical functioning, Role functioning, Pain, Social functioning, Emotional well-being, Energy/fatigue, Cognitive functioning, General Health, Health distress), and Overall QoL. These are scored as summary percentile scales ranging from 0 to 100, with higher scores indicating better health.

### Sleep.

The Pittsburgh Sleep Quality Index (PSQI)^38^ is a self-rated questionnaire that assesses sleep quality and disturbances over 1 month. It consists of 19 items combined to form seven “component” scores: subjective sleep quality, sleep latency, sleep duration, habitual sleep efficiency, sleep disturbances, use of sleeping medication, and daytime dysfunction. Each component is scored on a scale from 0 to 3, with 3 indicating the greatest dysfunction. The seven component scores are then summed to produce a global PSQI score, which ranges from 0 to 21, with higher scores indicating worse sleep quality.

### Social functioning.

The NIH Toolbox Social Satisfaction Factor Score measures aspects of social relationships, including emotional support, instrumental support, friendship, loneliness, and perceived rejection.^39,40^ This summary score represents an individual’s overall social satisfaction, with higher scores indicating greater satisfaction and lower scores suggesting potential areas of social distress.

*Activities of daily living* were evaluated using an adaptation of the Lawton-Brody instrumental activities of daily living (IADLs) scale that assessed self-reported changes in levels of independence in performing 16 everyday tasks, such as doing laundry and financial management, which are rated concerning whether the participant requires more assistance now than in the past. Each task of the IADL is scored as 0 (no change from best functioning) or 1 (more dependent now).^41^ The IADL total score ranges from 0 to 16 and is categorized overall as IADL independence (0–1) and IADL dependence (2–16), which signifies a need for help in accomplishing IADLs (IADLs impaired). We summed the IADL complaints on this instrument.

## Statistical methods

Table 1 summarizes the key predictors, modifiers/mediators, and outcomes in the current study. The statistical analysis compared the prevalence of chronic pain between people with HIV (PWH) and people without HIV (PWoH) using Fisher’s exact test, which is robust for small sample sizes. Odds ratios and 95% confidence intervals were calculated to quantify effect sizes. Secondary analyses examined associations between chronic pain and clinical measures, using Cohen’s d for continuous variables and Cramér’s V for categorical variables. Confidence intervals for effect sizes were generated through bootstrapping (10,000 iterations). For subgroup analyses with fewer than five participants, only descriptive statistics were reported, and no formal statistical tests were conducted. Demographic and clinical characteristics, including age, sex, race/ethnicity, education, HIV duration, CD4 counts, and viral suppression status, were summarized using appropriate measures of central tendency and variability (means, medians, and percentages).

We focused on modern statistical practices tailored for smaller sample sizes by prioritizing effect sizes and confidence intervals over p-values. For ordinal data in contingency tables, nonparametric methods were used, with chi-square and Cramér’s V statistics accompanied by bootstrapped confidence intervals. Quality of life was assessed through comparisons of the physical and mental health scores from the MOS-HIV. Associations between chronic pain and clinical measures, such as opioid use and interference in daily activities, were evaluated using logistic regression for binary outcomes and appropriate effect size metrics for continuous or categorical outcomes. Multivariable logistic regression was used to identify risk factors for chronic pain across demographic, HIV-specific, and medical domains, incorporating variables significant at p < 0.10 in univariate analyses. Log transformations were applied to non-normally distributed variables to ensure the validity of statistical assumptions. Adjustments for multiple comparisons, such as false discovery rate (FDR) correction, were applied where relevant to minimize type I error, particularly in analyses involving multiple outcomes, such as BDI-II subscales. The analyses described are based on variables with complete data for all participants, except the BDI-II), where 25 participants had missing data due to the COVID-19 context and ethical considerations surrounding remote data collection. To address concerns about selection bias, we compared chronic pain, demographic characteristics, and HIV disease variables between participants with and without BDI-II data.

## Results

### Participants

We evaluated 63 people, 40 PWH and 23 PWoH. Table 2 summarizes the demographic characteristics by HIV serostatus and HIV disease and treatment status of PWH; none of the demographic differences were statistically significant. Among PWH, the mean duration of HIV infection was 27.0 (9.46) years, the nadir CD4 + T lymphocyte count was 300, and the current CD4 + was 644; 100% took ART, and all were virally suppressed. Table 3 compares the demographic and HIV disease and treatment characteristics of PWH without CP (n = 16) and with CP (n = 24). Age, sex, and race/ethnicity were unrelated to CP. None of the HIV disease or treatment variables differed according to CP.

### Frequency of chronic pain and associated characteristics

#### Primary Analysis:

CP frequency was significantly higher in PWH (60%) than in PWoH (22%, odds ratio 5.4 [95% CI: 1.67, 17.5]; Fisher’s exact p = 0.0028). *Secondary analyses*: Among those who reported chronic pain, the average pain intensity did not differ by HIV status: ranged from 10 to 90 out of 100 (0 = no pain; 100 = worst possible pain) (mean+/− SD 53.1+/−22.6) for PWH versus 40 to 65 (51+/−11.4) for PWoH (Cohen’s d 0.109). Chronic pain interfered more with daily activities among PWH (Not at all 0.0%, A little 21.4%, Somewhat 35.7%, Quite a bit 14.3%, Very much 28.6%) than PWoH (33.3%, 66.7%, 0.0%, 0.0%, 0.0%) (chi-square statistic 8.85, Cramér’s V [95% CI] 0.552 [0.167, 0.714]). PWH with CP used analgesics and adjunctive pain medications for chronic pain more often than PWoH with CP (“Almost never” 12.5%, “Once in a while” 29.2%, “About half the time” 4.17%, “Frequently,” 12.5%, “Almost Always” 41.7% versus 80%, 20%, 0%, 0%; Cramer’s V = 0.612 [95% CI 0.146, 0.615]). Participants with CP more frequently used opioids (48.3%) than those without CP (20.6%) (OR 3.6 [1.19, 10.8].

## Chronic pain sources

**Figure 1:**
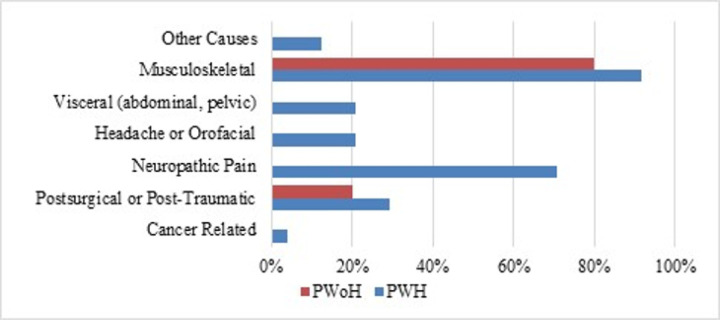
Self-reported sources of chronic pain in people living with and without HIV Note. PWH = people with HIV; PWoH = people without HIV.

**Figure 2:**
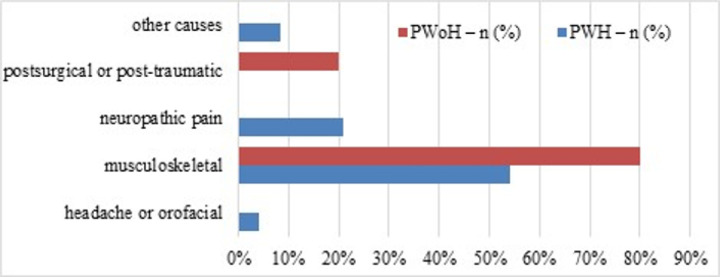
Self-reported primary source of pain in people living with versus without HIV. Note. PWH = people with HIV; PWoH = people without HIV.

Figure 1 shows the sources of chronic pain reported by study participants for PWH and PWoH. Note that participants could report multiple pain sources; among PWH with CP, 79.2% reported multiple sources. Chronic neuropathic pain was the only source category that differed between the two groups, affecting 70.8% of PWH versus 0% of PWoH. In both groups, musculoskeletal pain was the most frequent (80.0% versus 91.7%). The distribution of the other sources did not differ significantly between the two groups. Among PWH, the frequency of headache or orofacial pain was low, with only one individual affected, accounting for 4.2% of the cohort. Figure 2 illustrates the participant-reported *primary* source of pain among those with and without HIV. Neuropathic pain was the primary source in 5 PWH (20.8%), while other causes of pain were noted in only 2 participants (8.3%). Postsurgical or post-traumatic pain was reported as the primary source in none of the PWH CP group participants (vs 20% of their PWoH counterparts). PWoH reported no cases of a primary source of CP being headache or orofacial, neuropathic, or other causes. Musculoskeletal pain was reported as the primary source in 13 PWH (54.2%) versus 80% of the PWoH group. Among PWH, none reported postsurgical or post-traumatic pain as primary compared to 20% of PWoH.

## Relationship of chronic pain to depressed mood

**Figure 3:**
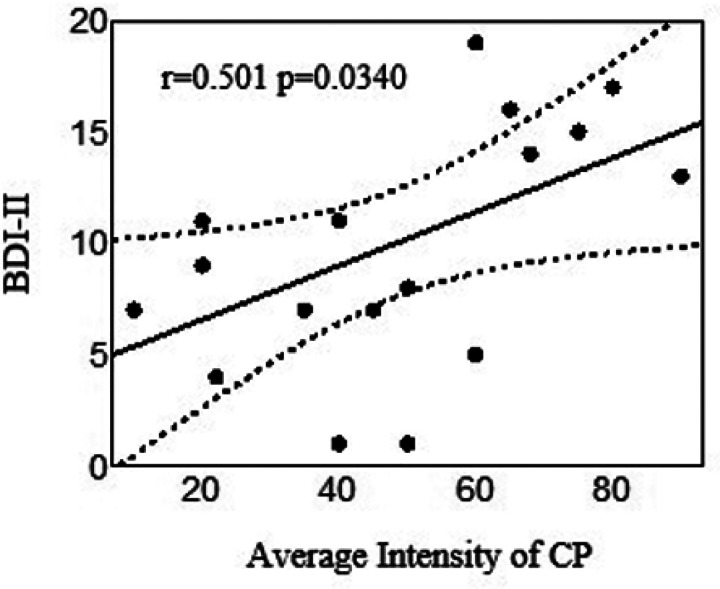
Relationship of chronic pain to depressed mood (total BDI-II score) in people living with HIV. Note. BDI-II = Beck Depression Inventory version 2, CP = chronic pain.

Figure 3 shows the relationship between chronic pain and depressed mood, indexed by the total BDI-II score, in PWH. PWoH are omitted due to the small sample size. BDI-II scores were missing for 25 participants because the data were collected remotely during the COVID-19 epidemic when ethical considerations dictated that participants were not asked about suicidal ideation if immediate in-person clinician intervention was not available. Given the small number of PWoH with both CP and BDI-II scores (n = 3), analyses of depression focused on PWH only. In PWH for whom data were available, BDI-II scores were significantly higher (worse depressed mood) in those with CP compared to those without (7.77 ± 5.86 vs 2.92 ± 3.06, Cohen’s d = 1.24). Increasing pain severity correlated with worse depressed mood in PWH (r = 0.501). Antidepressants were used by 45.0% of PWH; the association of chronic pain with antidepressant use was weak (OR 1.28 [0.45, 3.61]). Table 4 shows effect sizes for the relationships of chronic pain with the BDI-II subscales for PWH. PWH are omitted due to the small sample size. Effect sizes in PWH for the somatic, affective, apathy, and anhedonia subscales were moderate in size (0.36 to 0.41).

### Relationships between chronic pain and other clinical measures

Among PWH, CP was associated with worse physical (37.5±9.88 versus 52.5±9.73, Cohen’s *d* = 1.17) but not mental health-related QOL (MOS) scores (48.1±8.17 versus 55.0±6.45, Cohen’s *d* = 0.548). PWH had more IADL complaints than PWoH (median [IQR] 1 [0, 2] versus 0 [0, 0], Cohen’s d = 0.729), but among PWH, those with CP were no more likely those with and without CP did not differ in number of ADL complaints (1 [0.75, 4.25] versus 1 [0, 2], Cohen’s *d* = 0.362). PWH were less likely to be employed than PWOH (12 [63.2%] versus 10 [26.3%],). Among PWH, those with CP were not less likely to be employed than those without (6 [26.1% versus 4 [26.7%]). PWH had higher (worse) PSQI scores than PWoH (8.78±3.63 versus 6.84 ± 2.71, Cohen’s d = 0.563). Across all participants, PSQI scores were worse than those with chronic pain than those without (9.52 ± 3.48 versus 6.83 ± 2.92, Cohen’s *d* = 0.780). In a multivariable model including both HIV serostatus and chronic pain, only chronic pain was significantly associated with PSQI (partial ηp2s for HIV serostatus, CP, and their interaction, respectively, 0.0328, 0.0805, and 0.00579). PWH with CP had numerically but non-significantly lower (worse) mean NIH Toolbox Social Satisfaction Summary T Scores (41.3±13.4 versus 47.9±13.8; Cohen’s *d* = 0.493). In a multivariable analysis of the effects of chronic pain (CP) and depressed mood (BDI-II) on quality of life (QoL [MOS-HIV] in PWH) the interaction term was not significant (t Ratio 0.49). Chronic pain and depressed mood had an additive adverse effect on QoL (partial ηp2s 0.123, 0.180; model R^2^ 0.509).

PWH had a higher odds of polypharmacy than PWoH (OR 41.8 [95% CI 7.13, 808). In PWH, CP was not related to polypharmacy (OR 1.46 [0.381, 5.57). While people with HIV showed higher rates of antidepressant medication use than PWoH (n = 18 [45%] versus n = 4 [17.4%], OR 3.89 [1.12, 13.5]); PWH with CP did not differ from those without CP (n = 10 [41.7%] versus n = 8 [50%], OR 1.4 [0.392, 5.00]).

### Impact of potential biases related to missing BDI-II data

To address concerns about selection bias, we compared chronic pain, demographic characteristics, and HIV disease variables between participants with and without BDI-II data. Chronic pain rates did not differ for those with and without BDI-II data (44.7% versus 68.0%, OR 2.63 [0.913, 7.546]); nor did HIV serostatus (68.4% versus 56.0%, OR 1.7 [0.6, 4.84]). Participants without complete BDI-II data had a significantly higher median nadir CD4 count (243±179 versus 114±123, Cohen’s *d* = 0.826), while absolute CD4 counts were similar for the two groups (674±264 versus 646±204, Cohen’s *d* = 0.116). Viral suppression status, indicated by plasma viral load < = 50 copies/mL, showed high rates in both groups, with 97.7% of those with complete BDI-II data and 100% of those without. Demographic variables, including age (Cohen’s *d* = 0.0744), sex (odds ratio 1.08 [0.332, 3.49]), ethnicity (Cramer’s V = 0.249), and education (14.4±2.33 versus 14.4±2.20, Cohen’s d = 0.0022), did not show significant differences between groups. In a multivariable analysis adjusting for nadir CD4, rates of CP remained elevated in PWH compared to PWoH: For BDI-II available, OR 6.75 [1.43, 31.8]; for BDI-II not available, OR 3.38 [0.524, 21.7].

## Discussion

This exploratory analysis in a small cohort suggests substantially higher chronic pain frequency in virally suppressed PWH compared to PWoH, generating hypotheses for future research. This elevated prevalence aligns with prior studies, reinforcing the unique vulnerability of PWH to CP due to biological, psychosocial, and clinical factors. The findings also highlight CP as a major burden in PWH, with detrimental effects on daily functioning, mood, and quality of life.

One key finding is the significantly higher frequency of neuropathic pain among PWH, which likely contributes to their overall CP burden. Neuropathic pain, primarily associated with distal sensory polyneuropathy (DNP), was nearly absent in PWoH but prevalent in PWH. Neuropathic pain is known to be related to other adverse outcomes, such as depression and cognitive decline.^35,42,43^ This difference underscores the role of HIV-related pathophysiology, including chronic immune activation and neuroinflammation, in driving neuropathic pain.^44^

The relationship between CP and current depressed mood was pronounced in PWH. This finding is consistent with prior work showing shared neurobiological pathways involving inflammation, neurotransmitter dysregulation, and neural circuitry alterations linked to mood and pain.^7,45–47^ Astrocytes and microglia play roles in mood and antidepressant responses by reacting to neuronal damage and neurotransmitter imbalances.^48^ They coordinate responses to neuronal injury and neurotransmitter dysregulation through proliferation and the NLRP3 cytokines IL-1β and IL-18 secretion.^49,50^ Activation of both astrocytes and microglia has been demonstrated in HIV,^48^ and CP in PWH is associated with elevated levels of pro-inflammatory M1 macrophage chemokines.^51^ These shared mechanisms, compounded by the persistent inflammatory milieu in virally suppressed PWH, amplify the bidirectional relationship between CP and mood disorders. These insights underscore the importance of focusing research and interventions for DNP within the PWH population.

Overlapping anatomical pathways such as the brain’s reward system, which includes mesolimbic dopaminergic projections, may explain links between CP and depressed mood. Dopamine deficiency, common in PWH, is linked to the frequency of chronic pain and its association with depressed mood, particularly anhedonia.^52–54^ CP affects the neuronal pathway from the dorsolateral bed nucleus of the stria terminalis (dlBNST) to the ventral tegmental area (VTA), with stress-induced corticotropin-releasing factor (CRF) signaling leading to suppression of the mesolimbic dopaminergic system, contributing to depressed mood and CP.^54^ The serotonin (5-HT) and norepinephrine (NE) neurotransmitter systems, implicated in both CP and depressed mood, are believed to mediate the effects of most antidepressants used to treat both.^55^ These neurotransmitters are part of the endogenous analgesic system, suppressing pain afferents; thus, antidepressants may alleviate some pain conditions through the 5-HT system.^56,57^

Our study also highlights significant impairments in physical health-related quality of life among PWH with CP, confirming previous findings.^58,59^ CP in this cohort was associated with worse sleep quality and greater interference with daily activities. These findings emphasize the multidimensional impact of CP on physical, psychological, and social domains, necessitating integrated care approaches. The reliance on opioids and adjunctive pain medications among PWH with CP further underscores the challenges of pain management in this population. Given the risks of opioid dependence, adverse effects, and drug-drug interactions, multimodal pain management strategies tailored to the specific needs of PWH are imperative.^6^

Contrasting with PWoH, we found in PWH a high frequency of multiple concurrent comorbid sources of pain, including musculoskeletal, visceral (abdominal, pelvic), neuropathic pain, and postsurgical or post-traumatic.^1,3,4,14^ This issue is important because treating CP in PWH requires considering these multiple sources and the need for multidisciplinary, integrated care.

Interestingly, demographic factors such as age and sex did not show significant associations with CP in PWH, diverging from patterns observed in the general population. This discrepancy may reflect the complex interplay of HIV-specific factors, including accelerated aging and unique comorbidity profiles. The lack of association between CP and HIV-specific clinical variables such as CD4 counts and ART status suggests that CP in PWH is influenced more by chronic immune activation and comorbid conditions than by direct virologic control.

### Limitations

Statistical power in this study was limited by the relatively small sample sizes, particularly for PWoH, limiting statistical power and generalizability. However, our data align with established population prevalence estimates of 20–30% in PWoH, supporting the representativeness of our sample. Furthermore, this sample size is appropriate given the novelty and specific clinical nature of the cohort—individuals with HIV who are successfully treated with contemporary ART regimens. While our PWH sample size also was small, our findings align with previous literature regarding elevated CP frequency in PWH.^1–4^ The exploratory nature of the analyses and the absence of longitudinal data constrain causal inferences. Additionally, the reliance on self-reported measures for pain and mood introduces potential biases, although the use of validated instruments mitigates these. Participants’ self-reported sources of pain might not always align with clinical physio-anatomical diagnoses, potentially due to limited health literacy or the phenomenon of referred pain. This discrepancy, however, does not detract from the core objective of our study: to illuminate the patient’s lived experience of pain and their clinical associations. due to COVID-related ethical concerns about asking participants about suicidal ideation, BDI-II data was missing for some participants; however, additional analyses suggested that this was unlikely to bias the interpretation of mood-related outcomes. Females were underrepresented (24%) in our PWH sample. While this limited our ability to examine sex differences, our sample approximated the frequency of females among PWH in the United States (23%).^60^

### Implications and future directions

Our findings emphasize the clinical importance of a comprehensive, multidisciplinary approach to managing CP in PWH. Integrating thorough pain assessments into routine HIV care may help address the multifaceted impact of CP on mood, pain perception, and social functioning. Evidence supports the use of integrated pain management strategies, encompassing both pharmacological and non-pharmacological interventions, such as physical activity, which has shown protective effects against chronic pain. The observed reliance on analgesics and adjunctive medications among PWH highlights the need for careful medication management to minimize adverse effects and interactions with antiretroviral therapies.

Future research should prioritize larger, longitudinal studies to clarify causal pathways linking HIV, CP, and mood disorders. Incorporating biomarkers of inflammation and neuroimaging could shed light on underlying mechanisms and identify therapeutic targets. The observed reliance on analgesics, including opioids, and adjunct pain medications in our study population indicates the importance of careful medication management to avoid adverse effects and interactions, particularly with antiretroviral therapies. Evaluating multimodal pain management strategies, including physical activity therapy and cognitive-behavioral therapy, is essential. Additionally, investigating the social determinants of CP and mood disorders may inform interventions to reduce health disparities. Precision medicine approaches, such as genetic and epigenetic profiling, could optimize pain management, while digital health technologies offer the potential for real-time monitoring and dynamic treatment adjustments. These efforts, alongside the exploration of innovative treatments like neuromodulation, could significantly enhance the care of PWH with CP.

## Appendix

Supplementary Appendix A is not available with this version.

## Figures and Tables

**Table 1:** Key predictors, modifiers/mediators, and outcomes in the current study.

Predictors	Outcomes	Moderators/Mediators
Chronic Pain	Depressed mood	Demographics
Pain severity	Interference in daily activities	HIV disease characteristics
	Analgesic use	Medical Comorbidities
	Quality of life and social functioning	
	Sleep	

**Table 2:** Demographic and clinical characteristics of people living with and without HIV.

	Statistic	PWH (n = 40)	PWoH (n = 23)	*P*
Demographics				
Age	M (SD)	61.3 (10.5)	57.5 (17.1)	0.346
Sex, female - N (%)	n (%)	9 (23.7%)	9 (45%)	0.099
Education - mean (SD)	M (SD)	14.1 (2.39)	15.1 (2.37)	0.110
Race/Ethnicity				0.693
Black	n (%)	9 (26.5)	4 (22.2)	-
Hispanic	n (%)	6 (15.8)	4 (20)	-
Non-Hispanic White	n (%)	22 (64.7)	12 (66.7)	-
HIV Disease and Treatment				
Estimated Duration of HIV	mean (SD)	27.0 (9.46)	-	-
Nadir CD4* count	median (IQR)	300 (17, 123)	-	-
Current CD4	median (IQR)	644 (488, 786)	-	-
On ART	n (%)	40 (100%)	-	-
Virally suppressed	n (%)	40 (100%)	-	-

Notes. PWH = people with HIV; PWoH = people without HIV.

* CD4 + T lymphocytes/μl (median (IQR)).

**Table 3:** Demographic and clinical characteristics of people living with HIV according to chronic pain status.

	Statistic	No CP (n = 16)	CP (n = 24)	*p*
Demographics				
Age	M (SD)	58.5 (11.5)	62.1 (9.1)	0.301
Sex, female	n (%)	2 (13.3%)	7 (30.4%)	0.212
Years of education	M (SD)	14.5 (2.85)	13.8 (2.07)	0.545
Race/Ethnicity				0.421
Black	n (%)	2 (12.5%)	7 (29.1.3%)	-
Non-Hispanic White	n (%)	10 (62.5%)	13 (54.2%)	-
Hispanic	n (%)	3 (18.8%)	3 (12.5%)	-
Other	n (%)	1 (6.25%)	1 (4.17%)	-
HIV Disease and Treatment				
Estimated Duration of HIV	median (IQR)	31.6 (17.8, 35.6)	27.7 (21.7, 34.7)	0.481
Nadir CD4 + T lymphocytes/ul	median (IQR)	190 (16, 338)	57 (18, 200)	0.240
Current CD4 + T lymphocytes/ul	median (IQR)	700 (493, 853)	618 (463, 740)	0.395
On ART	n (%)	13 (81.3%)	20 (83.3)	0.866
Virally suppressed	n (%)	20 (83.3%)	-	

*Note*. CP = chronic pain; PWH = people with HIV; PWoH = people without HIV.

**Table 4:** Comparison of Beck Depression Inventory II subscales by chronic pain status for people living with HIV.

HIV serostatus	BDI-II subscale	Effect Size CP versus no CP	FDR p
**PWH**	**Somatic**	**0.391**	**0.003**
**PWH**	**Affective**	**0.406**	**0.014**
**PWH**	**Apathy**	**0.368**	**0.014**
**PWH**	**Anhedonia**	**0.357**	**0.014**
PWH	Cognitive	0.288	0.198
PWoH	Somatic	0.094	0.993
PWoH	Affective	-	0.999
PWoH	Apathy	0.065	0.993
PWoH	Anhedonia	0.074	0.993
PWoH	Cognitive	0.260	0.993

*Note*. BDI-II = Beck Depression Inventory version 2; FDR = false discovery rate multiple test correction; PWH = people with HIV. PWoH were omitted due to the small number of participants able to complete the BDI-II. Bolded rows represent statistically significant differences. Rows are ranked by significance values.

## Data Availability

The data that were analyzed are available from the National NeuroAIDS Tissue Consortium-CHARTER Data Coordinating Center (https://www.nntc.org/content/relationship-charter) upon request. The code supporting the findings of this study is available upon reasonable request to the authors. Data and code can be requested by contacting Dr. Ronald J. Ellis at roellis@health.ucsd.edu.
